# Adverse Maternal, Perinatal, and Neonatal Outcomes in Adolescent Pregnancies: A Case-Control Study

**DOI:** 10.34172/jrhs.2023.105

**Published:** 2023-03-30

**Authors:** Farnaz Mohammadian, Monireh Moharram Nejadifard, Shabnam Tofighi, Lida Garrosi, Behnaz Molaei

**Affiliations:** ^1^Department of Obstetrics and Gynecology, School of Medicine, Zanjan University of Medical Sciences, Zanjan, Iran; ^2^Department of Midwifery, School of Nursing and Midwifery, Zanjan University of Medical Sciences, Zanjan, Iran

**Keywords:** Health status, Pregnancy, Adolescent, Pregnancy in adolescence, Pregnancy outcome, Iran

## Abstract

**Background:** Despite the increase in the age of marriage, early marriage and subsequent adolescent pregnancy remain a serious problem in many regions and societies. Due to low evidence in this regard, this study was conducted to determine the health consequences of adolescent pregnancy.

**Study Design:** This was a case-control study.

**Methods: ** The present study was performed on pregnant women who were referred to Ayatollah Mousavi hospital of Zanjan for delivery in 2021. Pregnant women with gestational age less than 19 years were considered as the case group and those with gestational age between 19-35 years as the control group. The pregnancy outcomes on the mother and the neonate were obtained through the researcher-made checklist. Chi-square test and student’s t-test were used to compare variables between the two groups.

**Results:** In this study, 169 adolescent and 258 adult mothers were compared as the case and control groups, respectively. The mean age of cases and controls was 17.41±1.22 and 30.66±6.46 years, respectively. Cesarean delivery (34.5% vs. 23.67%, *P*=0.017) and anemia during pregnancy (16.28% vs. 10.7%, *P*=0.005) were significantly higher in the control group, while mood disorder after delivery was significantly higher in the case group (11.24% vs. 5.84%, *P*=0.04). The Apgar score 5 minutes after birth and birth weight were significantly higher in mothers of the control group (*P*<0.05).

**Conclusion:** The results demonstrated that adolescent mothers are more prone to postpartum depression, and babies born to these mothers are more prone to low birth weight and a low Apgar score. Therefore, adolescent pregnancy should be managed as a high-risk pregnancy.

## Background

 Adolescent pregnancy is a public health challenge that has a negative impact on the health of the mother and the child. It can be related to physical, social, educational, and economic factors.^1^ Adolescents’ pregnancy indicates pregnancy in the age range of 10-19 years,^2^ and its negative consequences include increased risk of preterm delivery, low birth weight babies, and infant death.^3^ Nearly 11% of all births are adolescent births with different special distribution rates worldwide.^4^ The rate of adolescent pregnancy is more common in developing and less developed countries. According to the results of the meta-analysis study on 24 countries, this rate was 18.8% in Africa.^5^ Approximately two-thirds of the births of adolescent women are unwanted, and an unwanted pregnancy makes the mother emotionally or financially unprepared for pregnancy and childbirth and predisposes her to postpartum depression.^1^ Adolescent pregnancy and childbirth are among the main obstacles to employment and continuing education so that the results of a study showed that only half of the adolescent mothers obtain a high school diploma.^6^

 The results of a meta-analysis on 38 studies demonstrated that preterm birth, early membrane rupture, anemia, low birth weight, and fetal distress were the most common effects of adolescent pregnancies. Moreover, in this meta-analysis, it was observed that childbirth by cesarean section, gestational diabetes, placenta previa, polyhydramnios, and macrosomia were less common among adolescents compared to adults.^7^ Socio-economic disparities such as poverty, low level of education, and inadequate family support cause these women to be more prone to *sexually transmitted infections*, hazardous abortions, and mother and neonate complications.^8^

 Based on the results of a national survey in Iran, about 13% and 2.9% of the girls in this age group were married and had a live birth, respectively.^9^ According to the director general of the social and cultural affairs of Zanjan province statements, about 37 000 cases of the marriage of children under 14 years of age have been registered in the country in 2021, which is noticeable in this province with 1400 cases. Despite the increase in the average age of marriage in the country, early marriage and subsequent adolescent pregnancy remain a serious problem in many parts of the country. Studies conducted on the consequences of adolescent pregnancy in Iran are limited, and little information is available in this regard. Therefore, this study aimed at determining the health consequence of adolescent pregnancy, including maternal, perinatal, and neonatal outcomes in women of Zanjan.

## Methods

 The present case-control study was conducted on pregnant women who were referred to Ayatollah Mousavi hospital of Zanjan, a city in northwest Iran, for delivery from March 2021 to February 2022. The non-probability consecutive sampling was used to select the study groups until completing the required sample size. Pregnant women whose gestational age was less than 19 and 19-35 years were considered as the case and control groups, respectively.

 The inclusion criteria included first pregnancy, age less than 35 years in the control group, fetal age at birth of more than 20 weeks, and fetal weight at birth of more than 500 g. On the other hand, unwillingness to participate in the study was considered an exclusion criterion. Moreover, women with underlying diseases such as cancer, diabetes, high blood pressure, and kidney failure were excluded from the study.

 According to the study of Santhya et al,^10^ the rate of abortion and stillbirth in women of a young age for marriage and premature birth was 175% and 8%, respectively; therefore, considering these parameters and power of 80% for the study and the error level of 5%, the total of 210 people in each group was considered for the implementation of the study. However, at the end of the study, it was impossible to include 210 women in the case group, thus the sample size in the control group was increased to maintain the power of the study.

 The Ethics Committee of Zanjan University of Medical Sciences approved the study (Ethic code ZUMS.REC.1399.212). Verbal ethical consent was obtained from the participants. Demographic and clinical information of the subjects was gathered through an interview with the patient, as well as considering the patient’s medical record in the hospital in the first days after delivery. In addition, the pregnancy outcome on the mother included eclampsia, pre-eclampsia, anemia, type of delivery, gestational age, postpartum bleeding, puerperal endometritis, urinary tract infections, pyelonephritis, pneumonia, breast infection, mastitis, and breastfeeding status of the neonate. For most women, the first weeks and months after childbirth are a time of emotional upheaval. Intense feelings of joy, exhaustion, fatigue, confusion, loneliness, disappointment, anger, fear, and happiness are all common. Therefore, we monitored these mood disorders for up to 6 weeks after delivery for all women by telephone contact through a trained midwife.

 In this study, the perinatal outcome was defined as the occurrence of any adverse outcome before 7 days of life. Adverse perinatal outcomes were defined as the presence of either of parameters such as stillbirth, low birth weight, preterm birth, admission to the neonatal intensive care unit (NICU), and first-minute birth asphyxia.

 Neonatal outcomes were neonate weight, intrauterine growth restriction (IUGR), land 1-minute and 5-minute Apgar, as well as health problems at birth and breastfeeding ability.

 The two studied groups were described using descriptive statistics, including the frequency tables, percentage, mean, and standard deviation. Quantitative and qualitative variables between the two study groups were compared using student’s *t* test and Chi-square test, respectively. Data were analyzed using SPSS (version 26), and the significance level of relationships was considered less than 0.05.

## Results

 In the present study, 169 adolescent and 258 adult mothers were compared as the case and control groups, respectively.The mean age of cases and controls were 17.41 ± 1.22 and 30.66 ± 6.46 years, respectively (*P* < 0.001), and the mean age at marriage was 13 ± 4.51 and 19.66 ± 5.13 years in the mentioned groups, respectively (*P* < 0.001). The case group had significantly lower menstruation age (11.09 ± 4.1 vs. 12.94 ± 2.35 years, *P* < 0.001), body mass index before pregnancy (22.7 ± 4.63 vs. 24.78 ± 5.52 kg/m^2^, *P* < 0.001), and age at first gestation (13.51 ± 5.63 vs. 21.87 ± 7.36 years, *P* < 0.001).

 Demographic characteristics of both case and control groups are presented in Table 1. Regarding the level of education, a higher percentage of women in the control group had a diploma and academic education (*P* < 0.001). In terms of parents’ education, there was no significant difference between the two groups. Based on the results, 26.4% of women in the control group were employed, while none of the women in the case group were an employee (*P* = 0.001). The percentage of living in rural areas was also significantly higher in the women of the case group (68.05% compared to 52.71%, *P* = 0.002). The desire to marry was significantly higher in the control group (*P* = 0.01). There was no significant difference between the two groups in terms of the experience of violence by the spouse, as well as the receipt of pregnancy care and supplements (*P* > 0.05).

**Table 1:** The demographic characteristics of women in the two case and control groups

**Characteristics**	**Case group (n=169)**		**Control group (n=258)**		***P*** ** value**
	**Number**	**%**	**Number**	**%**	
Education					0.001
Illiterate	2	1.18	8	3.10	
Primary school	62	36.69	74	28.68	
Guidance school	71	42.01	61	23.64	
High school	29	17.16	19	7.36	
Diploma	5	2.96	55	21.32	
Academic	0	0	41	15.89	
Father education					0.08
Illiterate	46	27.22	102	39.53	
Primary school	90	53.25	107	41.47	
Guidance school	19	11.24	26	10.08	
Diploma	11	6.51	15	5.81	
Academic	3	1.78	8	3.10	
Mother education					0.44
Illiterate	67	39.64	113	43.80	
Primary school	76	44.97	104	40.31	
Guidance school	20	11.83	27	10.47	
Diploma	6	3.55	10	3.88	
Academic	0	0	4	1.55	
Occupation					0.001
House keeper	169	100	247	95.74	
Employee	0	0	11	4.26	
Location					0.002
Urban	54	31.95	122	47.29	
Rural	115	68.05	136	52.71	
Marriage desire					0.01
Volunteer	148	87.57	244	94.57	
Compulsory	21	12.43	14	4.53	
Pregnancy tendency					0.29
Voluntarily	113	66.86	185	71.71	
Unwanted	56	33.14	73	28.2	
Experience of spouse violence					0.25
Yes	9	5.33	8	3.10	
No	160	94.67	250	96.90	
Accessing prenatal care					0.30
Yes	166	98.22	249	96.51	
No	3	1.78	9	3.49	
Accessing supplements					0.55
Yes	168	99.41	255	98.84	
No	1	0.59	3	1.16	

Table 2 compares pregnancy results between case and control groups. Cesarean delivery (34.5% vs. 23.67%, *P* = 0.017) and anemia during pregnancy (16.28% vs. 10.7%, *P* = 0.005) were significantly more considerable in control group mothers, while mood disorder after delivery was significantly higher in women of the case group (11.24% vs. 5.84%, *P* = 0.04). The Apgar score 5 minutes after birth (8.78 ± 0.78 vs. 8.62 ± 0.86, *P* = 0.04) and birth weight (514.62 ± 3094.97 vs. 2874 ± 579.53 grams) were significantly higher in mothers of the control group (*P* < 0.05). Finally, there was no significant difference between the two groups with regard to other variables such as eclampsia, pre-eclampsia, postpartum hemorrhage, episiotomy, endometritis, breastfeeding, mother’s hospitalization in ICU and newborn’s hospitalization in NICU, stillbirth, IUGR, and delivery age (*P* > 0.05).

**Table 2:** Comparison of pregnancy outcomes between cases and controls

**Categorical variables**	**Case group (n=169)**		**Control group (n=258)**		***P*** ** value**
	**Number**	**%**	**Number**	**%**	
Mode of delivery					0.017
Cesarean	40	23.67	89	34.50	
Vaginal	129	76.33	169	65.50	
Anemia during pregnancy					0.005
Yes	12	7.10	42	16.28	
No	157	92.90	216	83.70	
Preeclampsia during pregnancy					0.17
Yes	6	3.55	17	6.59	
No	163	96.45	241	93.41	
Eclampsia during pregnancy					0.16
Yes	0	0	4	1.55	
No	169	100	254	98.45	
Postpartum hemorrhage					0.52
Yes	11	6.51	13	5.04	
No	158	93.49	245	94.96	
ICU admission					0.99
Yes	1	0.59	2	0.78	
No	168	99.41	256	99.22	
Episiotomy					0.95
Yes	2	1.18	4	1.55	
No	167	98.82	254	98.45	
Endometritis					0.49
Yes	2	1.18	6	2.33	
No	167	98.82	252	97.67	
Mood disorder					0.04
Yes	19	11.24	15	5.81	
No	150	88.76	243	94.19	
Breastfeeding					0.97
Yes	163	96.45	249	96.51	
No	6	3.55	9	3.49	
Stillbirth					0.56
Yes	2	1.18	1	0.39	
No	167	98.82	257	99.1	
IUGR					0.89
Yes	9	5.33	13	5.04	
No	160	94.67	245	94.96	
NICU admission					0.09
Yes	36	21.30	36	13.95	
No	133	78.70	222	86.05	
**Continuous variables**	**Mean**	**SD**	**Mean**	**SD**	***P*** ** value**
Neonate weight (g)	2874	579.53	3094.97	514.62	0.001
Delivery age (wk)	37.89	5.29	37.57	2.57	0.4
Apgar score (min 5)	8.62	0.86	8.78	0.78	0.04

*Note. *SD: Standard deviation; NICU: Neonate intensive care unit; IUGR: Intrauterine growth restriction.

## Discussion

 This study investigated the effect of pregnancy at a young age on the mother and the neonate. The results demonstrated that adolescent mothers have a lower level of education, mostly living in rural areas; in addition, they all were housewives and engaged in no income-generating jobs. Adolescent mothers had a higher probability of normal delivery and less anemia during pregnancy. However, adolescent mothers were more likely to have mood disorders following delivery. Regarding neonate-related risk factors, neonates born to these mothers had a lower Apgar score and birth weight.

 Usynina et al reported that the probability of an Apgar score of less than 7 was higher in adolescent females.^11^ However, our findings showed that the five-minute Apgar score was significantly higher in adult mothers. This case most likely indicates that the age of the mother is effective on the Apgar score of the newborn. In this regard, Ogawa et al also concluded that the Apgar score among adolescent mothers was lower compared to 20-24-year-old women.^12^

 Zeck et al found that cesarean delivery in adolescent mothers is similar to the general population, while cesarean delivery is increasing in the general population.^13^ In our study, the rate of cesarean delivery in adolescent women was low, and adolescent mothers were more likely to have normal deliveries. Based on the results of Yoosefi Lebni et al, this could be due to the fear of adolescent mothers regarding cesarean delivery.^14^ In the study by Derme et al, the natural delivery rate was higher in adolescent women. Better function of uterine muscles and greater flexibility of the connective tissue can justify this issue.^15^ In some studies, low birth weight in adolescent mothers was mentioned as the reason for the decrease in the cesarean rate in these women.^16,17^ On the other hand, the higher incidence of low birth weight is a reflection of preterm birth; this is because the fetus has less time to grow and gain weight in this type of birth. Further, anatomical immaturity and continued growth of the mother may be biological obstacles to the development of the fetus. It is noteworthy that the method of delivery can be influenced by the physician’s policy and the mother’s request. Therefore, our findings may be different from those of previous studies.

 Our findings represented that there is no significant difference in the probability of postpartum hemorrhage in the two groups, while the results of Kawakita et al,^18^ showed that postpartum bleeding is more considerable in adolescent mothers. Lee et al reported that adolescent mothers are more exposed to inadequate prenatal care compared to adult mothers, which can increase the risk of health problems in adolescent mothers and infants.^19^

 The results of a study in India revealed that the use of contraceptive methods, as well as iron and folic acid supplements in adolescent women, was significantly lower, and some factors such as their lower level of education, lack of independence, lack of full awareness, and family pressure for fertility were associated with this low access.^20^ In the present study, there was no significant difference between the two groups in terms of the level of care and access to supplements, and this difference can be due to the development of the health network up to the level of rural areas and the very high sensitivity of the health levels in mother and child care. Moreover, the appropriate care of the hospital’s medical staff (gynecologists, midwives, and nurses of the gynecology and obstetrics department) in the prevention and timely treatment of postpartum hemorrhage can justify this finding.

 Based on the results of the present study, adolescent mothers had less anemia during pregnancy. The findings of a study in Brazil demonstrated that anemia is a public health problem among pregnant teenagers, and the risk of anemia is even greater than that of adult mothers. This is because more iron intake is necessary to support this particular state of rapid growth, which involves drastic biological changes.^21^ Appropriate compliance of adolescent women in taking supplements can also be another possible explanation for this result.

 In the current study, adolescent mothers mostly lived in rural areas, which is consistent with the results of a previous study,^22^ indicating that adolescent pregnancy is strongly related to social factors. Additionally, the fact that adolescent pregnancy happens more often in socially deprived societies, social factors themselves can affect the adequacy of prenatal care in teenagers. Amjad et al found a higher prevalence of poor pregnancy outcomes among socioeconomically disadvantaged adolescents compared to their affluent counterparts, suggesting that specific subgroups of adolescent mothers may be at high risk of poverty.^23^

 According to our findings, the rate of postpartum depression was higher in adolescent women, which is in line with the findings of previous studies, representing that transition from childhood to adulthood is naturally associated with relatively intense psychological and social pressures on adolescent development. Therefore, any additional stress such as that caused by early marriage and sexual violence increases psychosocial pressures. Playing the role of a wife and mother for a teenage girl as a wife exposes her to psychological harm and affects their relationships. Hence, early marriage threatens mental health and increases depression.^20^

 Considering that the two groups had significant differences in terms of some background variables, part of the differences related to pregnancy outcomes can be attributed to these differences, and it can be considered a limitation of the study. On the other hand, answers to some questions were based on the patient’s self-reports, which made the results prone to information distortion. Moreover, the lack of random selection of the study groups is another study limitation. However, examining multiple outcomes in the two investigated groups with a relatively acceptable sample size can be considered the strength of the present study.

**Highlights**

- Adolescent mothers are more prone to postpartum depression
- Babies born to adolescent mothers are more prone to low birth weight
- Babies born to adolescent mothers are more prone to a low Apgar score

## Conclusion

 The results revealed that adolescent mothers are more prone to postpartum depression, and neonates born to these mothers are more prone to low birth weight and a low Apgar score. Therefore, adolescent pregnancy should be managed as a high-risk pregnancy.

## Acknowledgements

 We would like to gratefully acknowledge the Deputy of Research and Technology of Zanjan University of Medical Sciences for funding the study. We would also like to gratefully acknowledge all the staff of Ayatollah Mousavi Hospital.

## Competing Interests

 The authors declare that they have no competing interests.

## Funding

 This study was financially supported by the Deputy of Research and Technology of Zanjan University of Medical Sciences.

## References

[1] Maness SB, Buhi ER, Daley EM, Baldwin JA, Kromrey JD (2016). Social determinants of health and adolescent pregnancy: an analysis from the national longitudinal study of adolescent to adult health. J Adolesc Health.

[2] Sedgh G, Finer LB, Bankole A, Eilers MA, Singh S (2015). Adolescent pregnancy, birth, and abortion rates across countries: levels and recent trends. J Adolesc Health.

[3] Karataşlı V, Kanmaz AG, İnan AH, Budak A, Beyan E (2019). Maternal and neonatal outcomes of adolescent pregnancy. J Gynecol Obstet Hum Reprod.

[4] Minjares-Granillo RO, Reza-López SA, Caballero-Valdez S, Levario-Carrillo M, Chávez-Corral DV (2016). Maternal and perinatal outcomes among adolescents and mature women: a hospital-based study in the north of Mexico. J Pediatr Adolesc Gynecol.

[5] Kassa GM, Arowojolu AO, Odukogbe AA, Yalew AW (2018). Prevalence and determinants of adolescent pregnancy in Africa: a systematic review and Meta-analysis. Reprod Health.

[6] Chandra A, Martinez GM, Mosher WD, Abma JC, Jones J. Fertility, family planning, and reproductive health of U.S. women: data from the 2002 National Survey of Family Growth. Vital Health Stat 23. 2005(25):1-160. 16532609

[7] Karaçam Z, Kizilca Çakaloz D, Demir R (2021). The impact of adolescent pregnancy on maternal and infant health in Turkey: systematic review and meta-analysis. J Gynecol Obstet Hum Reprod.

[8] Shawky S, Milaat W (2000). Early teenage marriage and subsequent pregnancy outcome. East Mediterr Health J.

[9] Eydipour P, Torkashvand Moradabadi M, Alimondegari M (2022). Socioeconomic correlations of pregnancy in Iranian girls aged under 19 years based on the 2016 census. Payesh.

[10] Santhya KG, Ram U, Acharya R, Jejeebhoy SJ, Ram F, Singh A (2010). Associations between early marriage and young women’s marital and reproductive health outcomes: evidence from India. Int Perspect Sex Reprod Health.

[11] Usynina AA, Postoev V, Odland J, Grjibovski AM (2018). Adverse pregnancy outcomes among adolescents in Northwest Russia: a population registry-based study. Int J Environ Res Public Health.

[12] Ogawa K, Matsushima S, Urayama KY, Kikuchi N, Nakamura N, Tanigaki S (2019). Association between adolescent pregnancy and adverse birth outcomes, a multicenter cross sectional Japanese study. Sci Rep.

[13] Zeck W, Walcher W, Tamussino K, Lang U (2008). Adolescent primiparas: changes in obstetrical risk between 1983-1987 and 1999-2005. J Obstet Gynaecol Res.

[14] Yoosefi Lebni J, Khalajabadi Farahani F, Solhi M, Ebadi Fard Azar F (2021). Causes and grounds of childbirth fear and coping strategies used by Kurdish adolescent pregnant women in Iran: a qualitative study. J Reprod Infertil.

[15] Derme M, Leoncini E, Vetrano G, Carlomagno L, Aleandri V (2013). Obstetric and perinatal outcomes of teenage pregnant women: a retrospective study. Epidemiol Biostat Public Health.

[16] Kurth F, Bélard S, Mombo-Ngoma G, Schuster K, Adegnika AA, Bouyou-Akotet MK (2010). Adolescence as risk factor for adverse pregnancy outcome in Central Africa--a cross-sectional study. PLoS One.

[17] Gupta N, Kiran U, Bhal K (2008). Teenage pregnancies: obstetric characteristics and outcome. Eur J Obstet Gynecol Reprod Biol.

[18] Kawakita T, Wilson K, Grantz KL, Landy HJ, Huang CC, Gomez-Lobo V (2016). Adverse maternal and neonatal outcomes in adolescent pregnancy. J Pediatr Adolesc Gynecol.

[19] Lee SH, Lee SM, Lim NG, Kim HJ, Bae SH, Ock M (2016). Differences in pregnancy outcomes, prenatal care utilization, and maternal complications between teenagers and adult women in Korea: a nationwide epidemiological study. Medicine (Baltimore).

[20] Nguyen PH, Scott S, Neupane S, Tran LM, Menon P (2019). Social, biological, and programmatic factors linking adolescent pregnancy and early childhood undernutrition: a path analysis of India’s 2016 National Family and Health Survey. Lancet Child Adolesc Health.

[21] Pinho-Pompeu M, Surita FG, Pastore DA, Paulino DSM, Pinto ESJL (2017). Anemia in pregnant adolescents: impact of treatment on perinatal outcomes. J Matern Fetal Neonatal Med.

[22] Khazaei S, Bashirian S, Bathaei A, Sadri M, Shirani F, Jenabi E (2022). Pregnancy outcomes in adolescents: a case-control study in the west of Iran. Curr Womens Health Rev.

[23] Amjad S, MacDonald I, Chambers T, Osornio-Vargas A, Chandra S, Voaklander D (2019). Social determinants of health and adverse maternal and birth outcomes in adolescent pregnancies: a systematic review and meta-analysis. Paediatr Perinat Epidemiol.

